# Fever Correlation with Erythrocyte Sedimentation Rate (ESR) and C-Reactive Protein (CRP) Concentrations in Patients with Isolated Polymyalgia Rheumatica (PMR): A Retrospective Comparison Study between Hospital and Out-of-Hospital Local Registries

**DOI:** 10.3390/life12070985

**Published:** 2022-06-30

**Authors:** Ciro Manzo, Marcin Milchert, Carlo Venditti, Alberto Castagna, Arvind Nune, Maria Natale, Marek Brzosko

**Affiliations:** 1 Rheumatologic Outpatient Ambulatory, Health District No. 59, Azienda Sanitaria Locale Napoli 3 Sud, 80065 Naples, Italy; maria.natale.int@alice.it; 2 Katedra Reumatologii i Chorób Wewnętrznych, Klinika Chorób Wewnętrznych Reumatologii Diabetologii Geriatrii i Immunologii Klinicznej PUM, 71-457 Szczecin, Poland; marcmilc@hotmail.com (M.M.); brzoskom@pum.edu.pl (M.B.); 3 Rheumatologic Outpatient Clinic Health District Campobasso, Via Ugo Petrella 1, 86100 Campobasso, Italy; carlo.venditti@alice.it; 4 Primary Care Department, Azienda Sanitaria Provinciale Catanzaro, 88068 Soverato, Italy; albertocastagna@tiscali.it; 5 Department of Rheumatology, Southport and Ormskirk Hospital NHS Trust, Southport PR8 6PN, UK; arvind.nune@nhs.net

**Keywords:** polymyalgia rheumatica, fever, low-grade fever, diagnostic delay, acute-phase reactants, retrospective study, comparison study, rheumatology

## Abstract

Background: Polymyalgia rheumatica (PMR) is the most common systemic inflammatory rheumatic disease affecting the elderly. Giant cell arteritis (GCA) is a granulomatous vasculitis affecting the aorta and its branches associated with PMR in up to 20% of cases. In recent studies based on university hospital registries, fever correlated with the erythrocyte sedimentation rate (ESR) but not with C-reactive protein (CRP) concentrations at the time of diagnosis in patients with isolated PMR. A long delay to a PMR diagnosis was suggested to explain this discrepancy, possibly caused by laboratory alterations (for instance, anemia of chronic disease type) that can influence only ESR. We performed a retrospective comparison study between the university hospital and two out-of-hospital public ambulatory databases, searching for any differences in fever/low-grade fever correlation with ESR and CRP. Methods: We identified all patients with newly diagnosed PMR between 2013 and 2020, only including patients who had a body temperature (BT) measurement at the time of diagnosis and a follow-up of at least two years. We considered BT as normal at <37.2 °C. Routine diagnostic tests for differential diagnostics were performed at the time of diagnosis and during follow-ups, indicating the need for more in-depth investigations if required. The GCA was excluded based on the presence of suggestive signs or symptoms and routine ultrasound examination of temporal, axillary, subclavian, and carotid arteries by experienced ultrasonographers. Patients with malignancies, chronic renal disease, bacterial infections, and body mass index (BMI) > 30 kg/m^2^ were excluded, as these conditions can increase CRP and/or ESR. Finally, we used the Cumulative Illness Rating Scale (CIRS) for quantifying the burden of comorbidities and excluded patients with a CIRS index > 4 as an additional interfering factor. Results: We evaluated data from 169 (73 from hospital and 96 from territorial registries) patients with newly diagnosed isolated PMR. Among these, 77.7% were female, and 61.5% of patients had normal BT at the time of diagnosis. We divided the 169 patients into two cohorts (hospital and territorial) according to the first diagnostic referral. Age at diagnosis, ESR, CRP, median hemoglobin (HB), and diagnostic delay (days from first manifestations to final diagnosis) were statistically significantly different between the two cohorts. However, when we assessed these data according to BT in the territorial cohort, we found a statistical difference only between ESR and BT (46.39 ± 19.31 vs. 57.50 ± 28.16; *p* = 0.026). Conclusions: ESR but not CRP correlates with fever/low-grade fever at the time of diagnosis in PMR patients with a short diagnosis delay regardless of HB levels. ESR was the only variable having a statistically significant correlation with BT in a multilevel regression analysis adjusted for cohorts (β = 0.312; *p* = 0.014).

## 1. Introduction

Polymyalgia rheumatica (PMR) is the most common systemic inflammatory rheumatic disease affecting the elderly, especially those between the ages of 70 and 80 [1,2,3,4]. Its typical presentation includes a sudden-onset of disabling pain in both the shoulders and pelvic girdles and morning stiffness lasting >45 min, associated with neck pain and raised acute-phase reactants (APRs) (specifically, erythrocyte sedimentation rate [ESR] and C-reactive protein [CRP] concentrations) [5,6,7,8].

Constitutional manifestations, such as fever, weight loss, general malaise, and loss of appetite, are present in some PMR patients [9,10]. On the other hand, fever or low-grade fever can be the onset manifestation in some PMR-mimicking diseases, such as infections and malignancies [11,12,13,14,15], or can be a warning signal for an association with a large-vessel vasculitis, named giant cell arteritis [GCA]). GCA, a granulomatous vasculitis affecting the aorta and its branches, may be associated with PMR in up to 20% of cases [16,17,18,19,20].

We retrospectively evaluated a potential association of fever/low-grade fever with ESR and CRP in patients with newly diagnosed, isolated PMR followed at the rheumatology clinic of the Pomeranian Medical University in Szczecin, Poland. We observed that fever/low-grade fever correlated with ESR but not with CRP [21]. A Belgian research group previously reported similar findings [22]. The possibility that fever/low-grade fever at diagnosis may correlate with ESR but not with CRP in PMR patients seemed surprising. Indeed, in these patients, fever is thought to be caused by some cytokines, most notably interleukin-6 and interleukin-1 (IL-6 and IL-1), strongly associated with a CRP increase. On the other hand, fever may itself induce a CRP increase [23,24]. The Belgian researchers suggested that a long delay to a PMR diagnosis might explain this discrepancy possibly caused by laboratory alterations (among these, anemia of chronic disease type) that can influence only ESR [22].

In order to verify this hypothesis, we performed a retrospective comparison study between databases with a significantly different diagnosis delay, searching for any differences regarding the correlation of fever/low-grade with ESR and CRP at the time of diagnosis in patients with isolated PMR.

## 2. Materials and Methods

We identified all patients with newly diagnosed PMR in the period 2013–2020 in Szczecin University Hospital and in two Italian (Sant’Agnello and Boiano) out-of-hospital public ambulatory local registries. The enrolled patients from the Szczecin local registry were the hospital cohort. The enrolled patients from the Sant’Agnello and Boiano local registries were the territorial cohort. We used the 2012 classification criteria proposed by the European League against Rheumatism and the American College of Rheumatology (EULAR/ACR) collaborative group [9]. 

To obtain comparable data, we used the same inclusion and exclusion criteria as we did in the previously reported Polish cohort. In particular, we only included patients with a body temperature (BT) measurement at the time of diagnosis of PMR. BT was normal if <37.2 °C. Routine tests for differential diagnostics were performed at both the time of diagnosis and during follow-ups, warranting more in-depth diagnostic investigations if required. GCA was excluded based on the presence of suggestive signs or symptoms and routine ultrasound examination of temporal, axillary, subclavian and carotid arteries by experienced ultrasonographers. We excluded patients with malignancies, chronic renal disease, and bacterial infections. We also excluded patients with body mass index (BMI) > 30 kg/m^2^, as this condition can increase CRP and/or ESR. Another exclusion criterion was a follow-up < two years to exclude diagnostic changes. Finally, we used the Cumulative Illness Rating Scale (CIRS) to quantify the burden of comorbidities and excluded patients with CIRS index > 4 as an additional interfering factor [25].

We divided all enrolled patients into two groups according to BT values: normal temperature and low-grade BT elevation/fever group. As per the study design, we first added and compared all the territorial data with the hospital ones, searching for possible differences. 

Statistical analysis: We recorded descriptive statistics for each variable and listed the quantitative variables as mean values and standard deviations or percentages. ANOVA test confirmed a normal distribution of observed variables, allowing to treat our sample with parametric tests. All *p*-values were two-sided, and a *p*-value of <0.05 was considered as statistically significant. Data analysis was performed using SPSS Statistics for Windows version 21 software (SPSS Inc., Chicago, IL, USA).

## 3. Results

Out of 556 patients with newly diagnosed PMR, we assessed data of 169 patients: 73 from hospital and 96 from territorial registries. Among these, 77.7% were female, and 61.5% had normal BT at the time of diagnosis.

After dividing all the enrolled patients into two groups according to BT values, we found a statistically significant difference only in the median ESR (54.25 ± 28.72 mm/h vs. 68.40 ± 31.81 mm/h; *p* = 0.002) (Table 1). A univariate analysis showed a statistically significant correlation between BT groups and ESR (r = 0.262; *p* = 0.006), confirmed by a subsequent stepwise multivariate analysis (β = 0.321; *p* = 0.001).

Following that, we divided the 169 patients into two cohorts (hospital and territorial) according to the first diagnostic referral. Age at diagnosis, ESR, CRP, median hemoglobin (HB), and diagnostic delay (days from first manifestations to final diagnosis) were statistically significant and different between the two cohorts (Table 2).

Age at diagnosis, ESR, CRP, and HB serum levels confirmed a statistically significant difference according to the BT class at the time of diagnosis of PMR in the two cohorts of patients (Table 3).

Interestingly, when we then assessed these data according to BT class in the territorial cohort, we found a statistical difference only between the ESR and BT classes (46.39 ± 19.31 vs. 57.50 ± 28.16; *p* = 0.026) (Table 4).

ESR was the only variable with a statistically significant correlation with BT in a multilevel regression analysis adjusted for cohorts (β = 0.312; *p* = 0.014).

## 4. Discussion

To the best of our knowledge, this is the first study to compare fever or low-grade fever with ESR and CRP at the time of diagnosis in patients with isolated PMR between university hospital and territorial registries. Our hospital and territorial PMR cohorts differ significantly. Regardless of HB level, ESR but not CRP correlates with fever/low-grade fever at the time of diagnosis and also in patients with a short diagnosis delay.

Fever and low-grade fever may be present at the time of diagnosis in patients with PMR, either alone or accompanied by other constitutional manifestations, such as weight loss, general malaise, loss of appetite, and fatigue. The literature has shown that fever and low-grade fever may be present in up to 50% of patients at the time of PMR onset [26,27]. We recorded fever/low-grade fever in 38.46% of patients at the time of PMR onset. 

Some researchers speculated that fever/low-grade fever and other constitutional manifestations are less common in PMR patients without significantly increased ESR [28]. More recently, these manifestations were infrequently reported in two large cohorts of PMR patients without elevated baseline APR (ESR and CRP) [29,30]. These observations indirectly imply fever/low-grade fever correlation with APR.

Fever is a shared finding in PMR and in some PMR-mimicking diseases, such as infections and malignancies, and a rapid but transient response to low-dosed systemic corticosteroids may be a confounding factor. Furthermore, some patients who were initially diagnosed with PMR are reclassified as having a different disease at their follow-ups. Therefore, a rigorous diagnostic process and an extended follow-up is mandatory to prevent misdiagnosis [31,32]. In particular, a follow-up <2 years is not recommended to rule out PMR as a paraneoplastic condition [33,34]. As a result, one of the inclusion criteria was a follow-up of at least 2 years.

PMR is commonly managed in general practice with a referral to a rheumatologist when diagnostic and/or therapeutic difficulties are present [35,36,37,38]. The Italian National Health System has a unique professional figure represented by the out-of-hospital public rheumatologist (OHPR), trained in intercepting patients before their referral to hospital or university clinics. A relatively short patient-waiting list is common in this setting. A short waiting list might favor a short diagnostic delay when compared to the Polish inpatient cohort. In addition, the OHPR is the only public specialist who visits PMR patients at home when their general practitioner (GP) requires a home visit. This is extremely beneficial in the management of PMR in the elderly and disabled [39]. Therefore, it is not surprising that certain characteristics of patients with PMR may differ based on the type of first referral. In particular, when we compared territorial with hospital cohorts (see Table 4), we observed that the age at diagnosis and HB serum levels were higher; CRP and ESR were significantly lower, and diagnostic delays were markedly shorter in the territorial cohort. However, we found no interference of fever/low-grade fever on CRP concentrations in the territorial cohort despite a short diagnosis delay. In other words, in our intercohort and intracohort assessments, the diagnostic delay was not a decisive factor in explaining the different interference of fever/low-grade fever on ESR and CRP.

Fever is more common in patients with GCA than PMR, and CRP levels in Horton patients with fever at onset may be within normal results [40,41,42,43,44,45,46]. As reported in our study design, experienced ultrasonographers performed routine US examinations of the temporal, axillary, subclavian, and carotid arteries, and suggestive US findings were used as the exclusion criteria [47,48,49]. Contrasted aortic computed tomography (CT) and 18-F fluorodeoxyglucose positron emission tomography (18-FDG PET) imaging were used instead only in some patients, generally due to a lack of consent by the patient or his/her family members. Therefore, we cannot rule out the possibility that some patients had an increased large-vessel FDG uptake at diagnosis. However, when comparing PMR patients with and without large-vessel FDG uptake, researchers found a similar proportion of patients with fever/low-grade fever [50,51]. As a result, this potential bias seemed a minor limitation of our study design. 

Are there any other fever-related factors that influence ESR more than CRP in patients with PMR? Specific data are currently nonexistent in the published literature, leaving only speculative hypotheses. We cannot rule out the possibility of using novel biomarkers in patients with isolated PMR who have a fever or a low-grade fever at the time of diagnosis. This could be an attainable target in future prospective studies.

Our study has limitations and strengths. The study design is a strength because inclusion and exclusion criteria were stringent. On the other hand, ours is a retrospective study which may be a limitation. Indeed, as in all retrospective studies, some laboratory data (for instance, fibrinogen, and IL-6 and IGs serum levels) may be missing. In addition, we cannot exclude the possibility that low-grade fever was not recorded in some patients. Lastly, as already highlighted, the Italian Health System provides a professional figure named OHPR, who is absent in other health systems. Therefore, differences in nonItalian health systems may make reproducibility of our study difficult. 

## 5. Conclusions

The correlation of fever or low-grade fever with ESR and CRP at the time of diagnosis in patients with isolated PMR is still controversial. According to our retrospective comparison study, ESR but not CRP correlates with fever/low-grade fever at the time of diagnosis also in patients with a short diagnosis delay.

Further prospective studies could pave the way for novel biomarkers associated with fever patterns in PMR patients, improving our understanding of the disease and enabling personalized management for these patients.

## Figures and Tables

**Table 1:** Erythrocyte sedimentation rate (ESR) and C-reactive protein (CRP) concentrations according to body temperature (BT) at the time of diagnosis in patients with isolated polymyalgia rheumatica (PMR).

	All (*n* = 169)	Normal Temperature < 37.2 °C (*n* = 104)	Low-Grade BT Elevation/Fever ≥ 37.2 °C (*n* = 65)	*p*
Age at diagnosis, years	71.17 ± 7.66	71.03 ± 7.95	71.41 ± 7.22	NS
Female, %	77.7%	76.9%	78.5%	NS
CRP, mg/L	30.88 ± 31.73	27.99 ± 31.36	35.50 ± 32.03	NS
ESR, mm/h	59.69 ± 28.72	54.25 ± 25.27	68.40 ± 31.81	0.002

Data expressed as mean ± standard deviation or percentage. NS = not significant.

**Table 2:** ESR, CRP, and HB median levels at the time of diagnosis of PMR based on hospital or territorial referrals.

	Hospital Cohort (*n* = 73)	Territorial Cohort (*n* = 96)	*p*
Age at diagnosis, years	68.69 ± 8.37	73.06 ± 6.50	<0.001
Female, %	75.3%	79.2%	NS
CRP, mg/L	46.73 ± 40.75	18.83 ± 13.53	<0.001
ESR, mm/h	72.31 ± 30.59	50.09 ± 23.11	<0.001
HB, g/dL	11.98 ± 1.40	12.80 ± 1.17	0.001
diagnostic delay = from first manifestations to diagnosis (days)	301.52 ± 716	24.3 ± 12.5	0.0002

Data expressed as mean ± standard deviation or percentage. NS = not significant.

**Table 3:** ESR, CRP, and HB serum levels according to BT class at the time of diagnosis of PMR in hospital and territorial cohorts.

	Normal Temperature (*n* = 104)			Low-Grade BT Elevation/Fever (*n* = 65)		
	Hospital Cohort (*n* = 40)	Territorial Cohort (*n* = 64)	*p*	Hospital Center (*n* = 33)	Territorial Center (*n* = 32)	*p*
Age at diagnosis, years	68.95 ± 9.18	72.33 ± 6.84	0.034	68.39 ± 7.41	74.58 ± 5.58	<0.001
Female, %	77.5%	76.6%	NS	72.7%	85.4%	NS
CRP, mg/L	45.70 ± 42.75	16.93 ± 12.36	0.000	47.97 ± 38.80	22.64 ± 15.10	<0.001
ESR, mm/h	66.82 ± 28.64	46.39 ± 19.31	0.000	78.97 ± 31.97	57.50 ± 28.16	<0.001
HB, g/dL	12.31 ± 1.16	12.93 ± 1.37	0.009	11.69 ± 1.52	12.54 ± 0.47	<0.001

Data expressed as mean ± standard deviation or percentage. NS = Not Significant.

**Table 4:** ESR, CRP, and HB according to BT at the time of diagnosis of PMR in our territorial cohort.

Territorial Cohort (*n* = 96)	Normal Temperature (*n* = 64)	Low-Grade BT Elevation/Fever (*n* = 32)	*p*
Age at diagnosis, years	72.33 ± 6.84	74.53 ± 5.58	NS
Female, %	77%	84.4%	NS
CRP, mg/L	16.93 ± 12.36	22.64 ± 15.10	NS
ESR, mm/h	46.39 ± 19.31	57.50 ± 28.16	0.026
HB, g/dL	12.93 ± 1.37	12.54 ± 0.47	NS

Data expressed as mean ± standard deviation or percentage. NS = Not Significant.
